# Effects of the selective orexin-2 receptor antagonist JNJ-48816274 on sleep initiated in the circadian wake maintenance zone: a randomised trial

**DOI:** 10.1038/s41386-021-01175-3

**Published:** 2021-10-09

**Authors:** Victoria L. Revell, Ciro della Monica, Jeewaka Mendis, Hana Hassanin, Robin J. Halter, Sandra R. Chaplan, Derk-Jan Dijk

**Affiliations:** 1 Surrey Sleep Research Centre, University of Surrey, Guildford, Surrey GU2 7XP UK; 2 Surrey Clinical Trials Unit, University of Surrey, Guildford, Surrey GU2 7XP UK; 3 Surrey Clinical Research Facility, University of Surrey, Guildford, Surrey GU2 7XP UK; 4 Janssen Research & Development, LLC, San Diego, CA USA; 5 UK Dementia Research Institute Care Research and Technology Centre, Imperial College London and the University of Surrey, Guildford, UK

**Keywords:** Circadian rhythms and sleep, Pharmacology

## Abstract

The effects of orexinergic peptides are diverse and are mediated by orexin-1 and orexin-2 receptors. Antagonists that target both receptors have been shown to promote sleep initiation and maintenance. Here, we investigated the role of the orexin-2 receptor in sleep regulation in a randomised, double-blind, placebo-controlled, three-period crossover clinical trial using two doses (20 and 50 mg) of a highly selective orexin-2 receptor antagonist (2-SORA) (JNJ-48816274). We used a phase advance model of sleep disruption where sleep initiation is scheduled in the circadian wake maintenance zone. We assessed objective and subjective sleep parameters, pharmacokinetic profiles and residual effects on cognitive performance in 18 healthy male participants without sleep disorders. The phase advance model alone (placebo condition) resulted in disruption of sleep at the beginning of the sleep period compared to baseline sleep (scheduled at habitual time). Compared to placebo, both doses of JNJ-48816274 significantly increased total sleep time, REM sleep duration and sleep efficiency, and reduced latency to persistent sleep, sleep onset latency, and REM latency. All night EEG spectral power density for both NREM and REM sleep were unaffected by either dose. Participants reported significantly better quality of sleep and feeling more refreshed upon awakening following JNJ-48816274 compared to placebo. No significant residual effects on objective performance measures were observed and the compound was well tolerated. In conclusion, the selective orexin-2 receptor antagonist JNJ-48816274 rapidly induced sleep when sleep was scheduled earlier in the circadian cycle and improved self-reported sleep quality without impact on waking performance.

## Introduction

The orexin (hypocretin) excitatory neuropeptides were first identified just over 20 years ago [1, 2] and have now been implicated in the regulation of a variety of functions and behaviours including the promotion of wakefulness [3]. The orexins A and B are produced by neurons of the lateral hypothalamus with major projections to wake-promoting regions including histamine cells of the tuberomammillary nuclei (TMN), noradrenergic cells of the locus coeruleus (LC), serotonergic cells of the dorsal raphe nuclei (DRN), and cholinergic cells in the basal forebrain (BF) [4]. The role of orexins in wake maintenance was discovered through the presence of disrupted orexin signalling in pre-clinical narcoleptic models and patients with narcolepsy [5, 6] who experience REM sleep intrusions during wake along with loss of muscle tone and cataplexy [7].

The contribution of the orexins to wakefulness is through activation of the Orexin-1 (OX1R) and Orexin-2 (OX2R) receptors in wake active structures [8]. The two orexin receptors differ in terms of their ligand affinity, expression and downstream effects. OX1R is expressed in the LC, OX2R in the TMN, whilst they are co-expressed in the DRN [2]. Orexin A shows similar affinity to both receptors whilst orexin B shows higher affinity to OX2R (reviewed in [3]). OX2R is found in all vertebrate genomes whereas OX1R is only found in mammals and is thus thought to have evolved from OX2R (reviewed in [3]). It has been proposed that activation of OX2R promotes wakefulness and suppresses NREM whilst activation of both receptors contributes to REM sleep suppression [9]. Orexinergic neurons show high neuronal firing activity preceding and during active wake whilst they are almost silent during non rapid eye movement (NREM) and REM sleep [10–12]. Furthermore, chemogenetic activation of orexinergic neurons results in REM sleep suppression [13].

The wake-promoting orexin system offers a treatment target for hypnotic pharmacological compounds. Such interventions can either target both receptors (dual orexin receptor antagonists, DORAs) or solely the OX2R (2-selective orexin receptor antagonists, 2-SORAs). Currently, two DORAs have been approved for use in insomnia disorder: suvorexant (Belsomra^®^; US and Japan) and lemborexant (Dayvigo^®^; US). However, pre-clinical and clinical studies have shown that selectively antagonising OX2R is sufficient to initiate and maintain sleep [8, 14] with minimal side effects [15].

Here we investigate the effects of JNJ-48816274, a selective, high-affinity 2-SORA, on sleep–wake regulation in a phase advance model of sleep disruption. In this model, sleep initiation is scheduled to coincide with the peak of the circadian wake propensity rhythm which is located in the evening hours (2–3 h before habitual bedtime). Sleep is rarely initiated in the early evening hours in this so-called wake maintenance zone [16], but if it is then sleep latencies are long [17]. Using this model we conducted a placebo-controlled, within-participant clinical trial that assessed the impact of two doses of JNJ-48816274 on sleep timing, structure, quality and spectral composition, as well as residual effects on cognitive performance, in healthy participants without sleep disorders.

## Materials and methods

### Study design and execution

This study was a randomised, double-blind, placebo-controlled, 3-period crossover evaluation of two different dose levels of the investigational medicinal product JNJ-48816274, performed as part of a larger clinical trial (study code 48816274EDI1001; Eudract number 2015-004186-89; ClinTrials.gov NCT02852395). The study used a 4-h phase advance model of sleep disruption where the beginning of the sleep opportunity was scheduled to occur 4 h before habitual bedtime in the wake maintenance zone [16], in which the drive for wakefulness is very strong [18]. This model has been used repeatedly to evaluate the effectiveness of putative hypnotics (e.g. [19–21]).

The study was conducted at a single site (Surrey Clinical Research Centre, University of Surrey, Guildford, UK) (June 2017-May 2018) and the protocol received a favourable ethical opinion (16/WA/0174) from Wales Research Ethics Committee 1, a National Research Ethics Committee. The study was conducted in accordance with Good Clinical Practice and the Declaration of Helsinki (2013).

### Study population and screening

All participants provided written informed consent prior to their participation in the study. Participants were healthy males aged 18–55 years inclusive, with a BMI between 18 and 30 kg/m^2^ and weighing ≥50 kg. Participants did not report any sleep–wake disorders or difficulty in falling or staying asleep. Participants could not consume >300 mg of caffeine per day or be a recent or current night shift worker or have travelled across >1 time zone within two weeks of their screening visit or during the study. Participants had to have a habitual bedtime between 22:00 and 00:00 h with a sleep duration of 7–8.5 h on the majority of days.

Potentially eligible participants completed a comprehensive medical screening and the Pittsburgh Sleep Quality Index (≤5) [22], STOP questionnaire (≤1) [23] and Epworth Sleepiness Scale (≤10) [24]. Participants also underwent an 8 h overnight clinical polysomnography (PSG) recording to confirm no presence of sleep disorders, acclimatise to the sleep laboratories, and for psychometric test battery training. From their screening visit until completion of the study, participants were requested to follow their habitual sleep/wake schedule, continually wearing an actiwatch (CamNTech, Cambridge, UK) and completing a daily sleep diary [25].

### Study design and treatments

The treatment phase consisted of three study periods (P1, P2, P3), each separated by a 7–9 day washout, and a follow-up visit (7–14 days after final dose). Participants received each of the three double-blinded treatments (placebo, 20 mg JNJ-48816274, 50 mg JNJ-48816274) as one of six computer-generated, randomisation sequences. The dose levels chosen were informed by the initial single ascending dose part of the trial. Each study period lasted ~48 h and included an adaptation night (Night −1, sleep scheduled at habitual time) and a treatment night (Night 1, sleep schedule advanced four hours e.g. a 23:00–07:00 h sleeper was in bed from 19:00 (Lights Off) – 03:00 h (Lights On)). On the P1 adaptation night only, participants were retrained on the test battery, their PSG was recorded and the test battery was performed upon awakening. These PSG and the morning test battery data were used as baseline measures for analysis.

On Night 1, participants received their study treatment (oral suspension) 15 min prior to Lights Off (participants could not eat in the 2 h prior to dosing). During their subsequent 8 h time in bed, PSG was recorded and the Centre’s through-the-portal system was used to draw regular pharmacokinetic blood samples without entering the room. Following Lights On, participants completed the Leeds Sleep Evaluation Questionnaire (LSEQ) [26], and Subjective Quality of Sleep Questionnaire (SQSQ) [27] followed by two psychometric test battery sessions (+8 h 45 min and +10 h 15 min post dose) to assess for any residual effects. Safety assessments were performed at discharge and follow-up.

### Sleep recordings

All sleep recordings (8 h time in bed) were obtained in individual sleep laboratories, which are sound attenuated, temperature and light-controlled with no windows. The sleep recording equipment used was the Somnomedics HD system with Domino software (v2.8), sampled at 256 Hz (S-Med, Redditch, UK) with an American Academy of Sleep Medicine (AASM) standard adult EEG montage. All sleep recordings were scored in 30 s epochs by an experienced sleep technologist in accordance with AASM guidelines (2016) [28]. EEG power spectrum analysis was conducted after artefact removal, for all nights that had sufficient data available. EEG power spectra were computed on 4 s epochs with 25% overlap between consecutive epochs; for each 30 s epoch an average power spectrum was computed (see [27] for description of spectral analysis procedures). For each 1 Hz bin, between 1 and 32 Hz, the mean of the 0.25 Hz data was calculated (e.g. for 1 Hz, the mean of 0.25, 0.50, 0.75 and 1 Hz was computed). In addition, eight band intervals were defined and the sum of power in each band interval was calculated: (1) 0.25 to <0.75 Hz, (2) 0.75 to <4.75 Hz, (3) 4.75 to <8 Hz, (4) 8.0 to <12.0 Hz, (5) 12.0 to <15.0 Hz, (6) 15.0 to <20.0 Hz, 7) 20.0 - <25.0 Hz, 8) 25.0 to <32.0 Hz (sum of 25.00–31.75 Hz). Data from the C3-M2 channel were used and if this was unavailable then C4-M1 was substituted.

### Performance testing

The performance test battery used to assess morning residual effects at treatment visits included both paper-based and electronic tests administered in the following order: Karolinska Sleepiness Scale (KSS) [29], Bond & Lader visual analogue scales (VAS) [30], body sway (to detect drug-induced changes in balance (e.g. [31]), Digit Symbol Substitution Test (DSST) [32], two Reaction Time tasks (e.g. [33]), Verbal N-back (1, 2 and 3) [34], and repeat KSS.

### Statistical Analysis

Study endpoints were separately analysed as dependent variables in a General Linear Mixed model using the mixed procedure (PROC MIXED) in SAS^®^ software with treatment, period and treatment by period interaction as independent categorical fixed effects, participant as random effect and the baseline night (P1 adaptation night) as a covariate. Analyses were conducted on an Intention to Treat Population defined as all randomised participants who received at least one dose and had both baseline and one post-baseline efficacy endpoint. Dependent variables were logarithmically (base 10) transformed prior to analysis if this was determined to be necessary following visual inspection of a plot of ranked normal-transformed model residuals. The sample size (*n* = 18) was considered adequate for initial evaluation of sleep in healthy participants.

The following sleep variables were analysed: (1) total sleep time (TST) (whole night and thirds of the night) (2) measures of sleep initiation and continuity: latency to persistent sleep (LPS), sleep efficiency (SE), sleep onset latency (SOL), wake after sleep onset (WASO), number of awakenings (NAW) (whole night and thirds of the night, as appropriate), duration of wake (3) sleep architecture: duration (min) and % of TST for sleep stages N1, N2, N3 and REM sleep for the whole night and thirds of the night, (4) latency to REM sleep (from sleep onset), (5) sleep EEG power spectra: separate analyses for each of the 32 ×1 Hz-width bins, for thirds of the night and the whole night, separately for NREM and REM sleep, (6) subjective sleep measures (LSEQ and SQSQ).

For the performance test battery, all variables were used as the dependent variable in a General Linear Mixed model (SAS PROC MIXED) with treatment, period, timepoint and treatment by period interaction as fixed effects, the P1 adaptation night morning measurements as the baseline covariate, participant as random effects and time as a repeated measure with unstructured variance covariance matrix. In most cases, compound symmetry covariance structure was used in place of unstructured matrix when convergence became an issue.

For the primary endpoints (TST and LPS), the hypothesis testing was one-sided at the 10% significance level. For all other endpoints, hypothesis testing was two-sided with 5% level of significance.

## Results

### Participant disposition

One hundred twenty-five healthy men were consented for the study, 70 underwent a PSG screening and, of those, 18, aged 28.9 ± 9.8 years (mean ± SD), were randomised into the study and completed all visits as planned.

### Datasets

Full datasets were obtained for analysis for all variables except EEG power spectra (16/18), DSST (16/18) and body sway (13/18).

### Pharmacokinetic profile

**Fig. 1:**
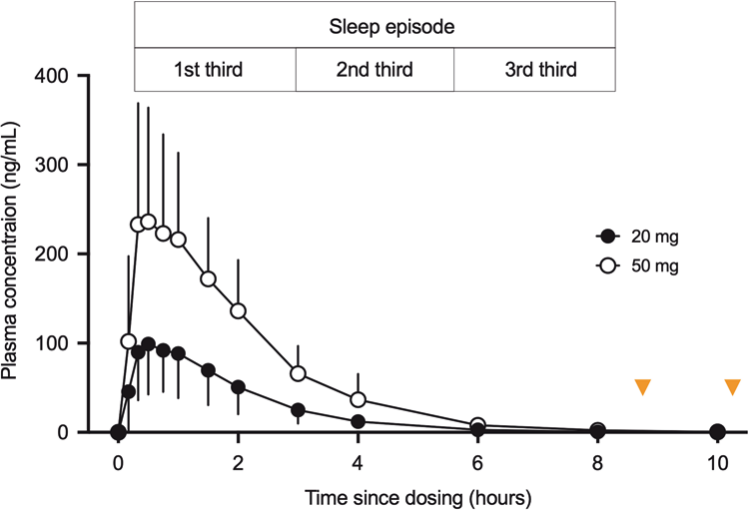
Pharmacokinetic profiles. Plasma concentrations (mean ± SD) of JNJ-48816274 following dosing with either 20 mg (black circles) or 50 mg (open circles) 15 min before bedtime. Orange triangles indicated the timing of the psychometric test battery in the morning.

The 20 mg dose had a Tmax of 0.58 ± 0.35 h, Cmax of 113 ± 52.2 ng/mL, and *t* ½ of 0.94 ± 0.18 h, and the 50 mg dose had a Tmax of 0.69 ± 0.49 h, Cmax of 269 ± 126 ng/mL, and *t* ½ of 0.96 ± 0.18 h (Fig. 1). Plasma concentrations were high in the first third, already diminishing in the second third, and near zero in the last third of the 8 hour sleep episode such that, upon awakening plasma levels of JNJ-48816274 were negligible (<3 ng/ml).

### Polysomnography—objective sleep measures

**Fig. 2:**
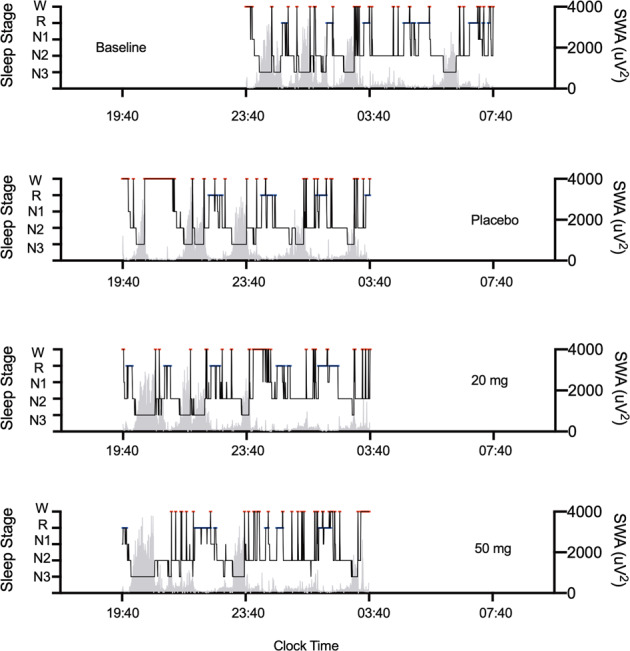
Hypnograms during baseline and three treatment nights. Representative hypnograms for participant 2018 indicating the time course of sleep stages across the night with associated slow wave activity (SWA) plots.

Sleep variables under the baseline and three treatment conditions were summarised for the whole night (Table 1a) and per thirds of the night (Table 1b). As exemplified in Fig. 2, when sleep was displaced 4 h earlier, under placebo there was an increase in time awake and decrease in time spent in N3 and slow wave activity (SWA), particularly in the first third of the night. With JNJ-48816274, N3 and SWA in the early part of the night were restored and similar to baseline.

**Table 1:** Sleep variables (mean + 95% confidence intervals) and differences between JNJ-48816274 treatment (20 and 50 mg) and placebo for the baseline and three treatment nights for (a) whole night, (b) per thirds of the night.

**(a)**														
**Sleep variable**	**Mean (95% CI)**								**Estimated Mean difference vs Placebo (*p*** **value)** *Estimated ratio of means between doses &Placebo (*p* **value)**				**Standard Mean Difference vs Placebo**	
	**Baseline**		**Placebo**		**20** **mg**		**50** **mg**		**20** **mg**		**50** **mg**		**20** **mg**	**50** **mg**
	**Mean**	**95% Confidence intervals**	**Mean**	**95% Confidence intervals**	**Mean**	**95% Confidence intervals**	**Mean**	**95% Confidence intervals**	Difference *ratio	***p*** **value**	Difference *ratio	***p*** **value**	**Difference**	**Difference**
Primary endpoints														
Latency to persistent sleep (min)*	6.51	(3.99, 10.63)	16.11	(10.48, 24.77)	5.22	(3.19, 8.56)	4.36	(2.65, 7.19)	0.32	0.00	0.27	0.00		
Total sleep time (min)	443.86	(431.91, 455.82)	396.22	(368.08, 424.36)	423.78	(399.70, 447.85)	423.28	(401.71, 444.85)	27.56	0.01	27.06	0.02	0.52	0.54
Secondary endpoints														
Sleep Onset Latency (min)*	4.51	(2.77, 7.33)	9.69	(6.32, 14.84)	3.40	(2.38, 4.85)	2.29	(1.57, 3.35)	0.35	0.00	0.24	0.00		
Stage N1 duration (min)*	33.20	(25.51, 43.12)	33.96	(27.45, 42.00)	29.71	(22.12, 39.89)	27.95	(21.31, 36.66)	0.88	0.34	0.82	0.17		
% of TST for N1*	7.49	(5.66, 9.92)	8.67	(6.97, 10.77)	7.06	(5.23, 9.55)	6.64	(5.00, 8.80)	0.82	0.16	0.77	0.07		
Stage N2 duration (min)	229.25	(217.58, 240.92)	215.86	(196.23, 235.50)	228.22	(206.65, 249.80)	232.39	(212.54, 252.24)	12.36	0.22	16.53	0.10	0.30	0.42
% of TST for N2	51.69	(49.23, 54.16)	54.40	(51.85, 56.94)	53.60	(50.15, 57.06)	54.73	(51.36, 58.10)	−0.80	0.66	0.33	0.85	0.13	0.06
Stage N3 duration (min)	85.22	(69.53, 100.91)	76.31	(62.66, 89.95)	76.94	(62.11, 91.78)	77.08	(65.46, 88.71)	0.64	0.90	0.78	0.88	0.02	0.03
% of TST for N3	19.09	(15.68, 22.50)	19.58	(16.08, 23.07)	18.34	(14.71, 21.97)	18.39	(15.45, 21.33)	−1.23	0.35	−1.18	0.37	0.17	0.18
REM duration (min)	91.22	(78.38, 104.07)	66.86	(56.43, 77.30)	82.92	(72.91, 92.92)	82.08	(72.38, 91.78)	16.06	0.01	15.22	0.02	0.78	0.75
% of TST for REM	20.44	(17.77, 23.11)	16.53	(14.25, 18.80)	19.52	(17.50, 21.53)	19.31	(17.48, 21.13)	2.99	0.02	2.78	0.04	0.69	0.67
NREM duration (min)	352.64	(340.92, 364.36)	329.36	(308.39, 350.34)	340.86	(320.09, 361.63)	341.19	(324.09, 358.30)	11.50	0.19	11.83	0.17	0.27	0.31
REM sleep latency (min)	100.72	(69.98, 131.47)	148.86	(109.26, 188.46)	92.28	(63.79, 120.76)	46.56	(24.13, 68.98)	−56.58	0.00	−102.31	0.00	0.82	1.58
% REM sleep latency ≤ 15 min					17%		44%							
Sleep efficiency (%)	92.47	(89.98, 94.96)	82.55	(76.69, 88.41)	88.29	(83.27, 93.30)	88.18	(83.69, 92.68)	5.74	0.01	5.64	0.02	0.52	0.54
Total time awake from SOL (min)*	20.97	(13.65, 32.22)	53.16	(37.65, 75.06)	35.61	(24.24, 52.33)	30.71	(19.86, 47.49)	0.67	0.04	0.58	0.01		
Number of night awakenings after LPS*	19.52	(15.34, 24.83)	24.29	(21.44, 27.51)	22.62	(18.79, 27.23)	19.68	(16.04, 24.15)	0.93	0.50	0.81	0.05		
Wake after sleep onset (min)*	21.84	(14.08, 33.86)	53.70	(37.73, 76.42)	38.35	(26.44, 55.63)	36.73	(22.58, 59.77)	0.71	0.08	0.68	0.05		

(b)															
Sleep variable	Analysis period	Mean (95% CI)								Estimated difference vs placebo (p value)				Standard mean difference vs placebo	
		Baseline		Placebo		20 mg		50 mg		20 mg		50 mg		20 mg	50 mg
		Mean	95% Confidence intervals	Mean	95% Confidence intervals	Mean	95% Confidence intervals	Mean	95% Confidence intervals	Difference	*p* value	Difference	*p* value	Difference	Difference
Primary endpoints															
Total sleep time (min)	First third of the night	147.94	(143.67, 152.22)	109.19	(88.27, 130.12)	148.50	(144.82, 152.18)	151.25	(147.19, 155.31)	39.31	0.00	42.06	0.00	1.30	1.39
	Second third of the night	153.78	(151.27, 156.29)	140.22	(126.98, 153.46)	142.08	(130.70, 153.47)	146.44	(138.42, 154.47)	1.86	0.76	6.22	0.31	0.07	0.28
	Third third of the night	142.14	(134.06, 150.22)	146.81	(142.81, 150.81)	133.19	(114.33, 152.06)	125.58	(107.15, 144.02)	−13.61	0.15	−21.22	0.03	0.50	0.79
Secondary endpoints															
Stage N1 duration (min)*	First third of the night	7.94	(5.77, 10.92)	11.42	(8.28, 15.74)	6.41	(4.03, 10.22)	6.32	(3.98, 10.03)	0.56	0.01	0.55	0.01		
	Second third of the night	7.39	(5.24, 10.44)	8.35	(6.11, 11.40)	9.84	(7.18, 13.48)	8.01	(5.37, 11.93)	1.18	0.40	0.96	0.83		
	Third third of the night	16.21	(12.31, 21.35)	11.80	(8.71, 15.98)	9.92	(6.05, 16.26)	10.79	(8.22, 14.17)	0.84	0.45	0.91	0.70		
% of TST for N1*	First third of the night	0.05	(0.04, 0.08)	0.12	(0.08, 0.18)	0.04	(0.03, 0.07)	0.04	(0.03, 0.07)	0.37	0.00	0.36	0.00		
	Second third of the night	0.05	(0.03, 0.07)	0.06	(0.04, 0.09)	0.07	(0.05, 0.10)	0.06	(0.04, 0.08)	1.15	0.51	0.90	0.63		
	Third third of the night	0.12	(0.08, 0.16)	0.08	(0.06, 0.11)	0.10	(0.06, 0.16)	0.09	(0.07, 0.12)	1.21	0.38	1.14	0.55		
Stage N2 duration (min)	First third of the night	66.53	(57.72, 75.34)	53.25	(40.83, 65.67)	63.83	(57.21, 70.45)	70.69	(63.56, 77.83)	10.58	0.03	17.44	0.00	0.53	0.86
	Second third of the night	89.67	(81.61, 97.72)	80.89	(69.38, 92.40)	83.31	(70.86, 95.75)	84.75	(75.31, 94.19)	2.42	0.70	3.86	0.53	0.10	0.18
	Third third of the night	73.06	(61.71, 84.40)	81.72	(76.67, 86.78)	81.08	(67.09, 95.08)	76.94	(62.87, 91.02)	−0.64	0.93	−4.78	0.51	0.03	0.22
% of TST for N2	First third of the night	0.45	(0.39, 0.51)	0.49	(0.43, 0.55)	0.43	(0.38, 0.48)	0.47	(0.42, 0.51)	−0.06	0.04	−0.02	0.43	0.52	0.20
	Second third of the night	0.59	(0.53, 0.64)	0.58	(0.51, 0.65)	0.59	(0.52, 0.65)	0.58	(0.53, 0.62)	0.01	0.81	0.00	0.96	0.06	0.02
	Third third of the night	0.51	(0.44, 0.58)	0.56	(0.52, 0.59)	0.57	(0.49, 0.66)	0.60	(0.51, 0.69)	0.02	0.76	0.04	0.40	0.11	0.32
Stage N3 duration (min)	First third of the night	57.89	(44.39, 71.39)	34.22	(24.03, 44.41)	57.06	(44.49, 69.63)	51.11	(43.03, 59.20)	22.83	0.00	16.89	0.00	0.99	0.91
	Second third of the night	18.25	(12.78, 23.72)	27.11	(17.75, 36.48)	13.75	(5.37, 22.13)	16.50	(10.48, 22.52)	−13.36	0.01	−10.61	0.04	0.75	0.67
	Third third of the night	9.08	(3.87, 14.30)	14.97	(8.70, 21.25)	6.14	(1.70, 10.58)	9.47	(4.69, 14.25)	−8.83	0.01	−5.50	0.12	0.81	0.49
% of TST for N3	First third of the night	0.39	(0.30, 0.48)	0.30	(0.23, 0.37)	0.38	(0.30, 0.46)	0.34	(0.29, 0.39)	0.08	0.03	0.04	0.26	0.56	0.33
	Second third of the night	0.12	(0.08, 0.15)	0.20	(0.13, 0.26)	0.10	(0.04, 0.16)	0.11	(0.07, 0.16)	−0.10	0.00	−0.08	0.02	0.78	0.73
	Third third of the night	0.06	(0.03, 0.10)	0.10	(0.06, 0.15)	0.04	(0.01, 0.07)	0.10	(0.02, 0.18)	−0.06	0.10	0.00	0.96	0.83	0.02
REM duration (min)	First third of the night	13.47	(9.62, 17.32)	8.33	(3.93, 12.74)	18.00	(11.52, 24.48)	20.58	(16.36, 24.80)	9.67	0.01	12.25	0.00	0.87	1.41
	Second third of the night	36.64	(29.21, 44.07)	22.36	(15.89, 28.83)	33.22	(25.97, 40.48)	34.78	(26.85, 42.71)	10.86	0.02	12.42	0.01	0.79	0.85
	Third third of the night	41.11	(31.58, 50.64)	36.17	(29.61, 42.72)	31.69	(23.75, 39.64)	26.72	(18.08, 35.36)	−4.47	0.37	−9.44	0.06	0.31	0.61
% of TST for REM	First third of the night	0.09	(0.06, 0.12)	0.06	(0.03, 0.10)	0.12	(0.08, 0.17)	0.14	(0.11, 0.16)	0.06	0.02	0.07	0.00	0.73	1.17
	Second third of the night	0.24	(0.19, 0.28)	0.15	(0.11, 0.19)	0.23	(0.18, 0.27)	0.24	(0.19, 0.29)	0.08	0.01	0.09	0.01	0.86	0.92
	Third third of the night	0.29	(0.22, 0.35)	0.25	(0.20, 0.29)	0.22	(0.17, 0.27)	0.20	(0.13, 0.26)	−0.02	0.48	−0.05	0.13	0.24	0.48
NREM duration (min)	First third of the night	134.47	(130.01, 138.94)	100.86	(82.16, 119.56)	130.50	(122.86, 138.14)	130.67	(126.26, 135.08)	29.64	0.00	29.81	0.00	1.03	1.09
	Second third of the night	117.14	(110.98, 123.29)	117.86	(107.92, 127.80)	108.86	(100.18, 117.55)	111.67	(102.06, 121.27)	−9.00	0.11	−6.19	0.27	0.48	0.32
	Third third of the night	101.03	(92.08, 109.98)	110.64	(104.68, 116.60)	101.50	(86.54, 116.46)	98.86	(84.74, 112.99)	−9.14	0.23	−11.78	0.12	0.40	0.54
Number of awakenings from sleep onset*	First third of the night	4.41	(3.46, 5.62)	7.20	(5.30, 9.79)	4.97	(3.36, 7.36)	3.98	(2.73, 5.81)	0.69	0.06	0.55	0.00		
	Second third of the night	6.03	(4.49, 8.10)	7.08	(5.41, 9.25)	8.81	(6.71, 11.57)	7.28	(5.82, 9.09)	1.25	0.09	1.03	0.82		
	Third third of the night	8.74	(6.50, 11.74)	10.00	(8.32, 12.03)	7.54	(5.49, 10.35)	8.06	(6.32, 10.28)	0.75	0.12	0.81	0.23		
Duration of Stage W (min)*	First third of the night	9.38	(6.38, 13.78)	33.77	(20.38, 55.93)	9.30	(6.55, 13.21)	6.51	(4.48, 9.47)	0.28	0.00	0.19	0.00		
	Second third of the night	4.81	(3.31, 6.98)	10.64	(6.20, 18.24)	10.00	(5.90, 16.95)	8.14	(4.98, 13.30)	0.94	0.82	0.77	0.33		
	Third third of the night	11.04	(6.38, 19.12)	11.24	(8.41, 15.04)	14.64	(8.87, 24.15)	17.35	(9.14, 32.93)	1.30	0.40	1.54	0.17		

For those variables that have been log transformed (indicated by *), the ratio of dose: placebo is reported rather than the difference between the dose and placebo.

### Effect of the phase advance model

**Fig. 3:**
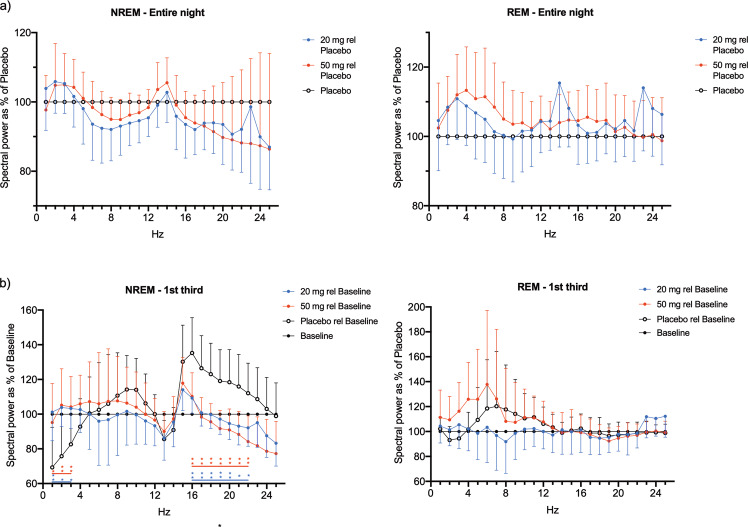
EEG power density during NREM and REM sleep. EEG power density (mean and 95% confidence intervals) **a** averaged across the entire night and expressed relative to placebo, and **b** averaged across the first third of the night and expressed relative to baseline (blue circles indicate 20 mg, red circles indicate 50 mg, black circles indicate baseline, open circles indicate placebo). Horizontal lines indicate significant differences between JNJ-48816274 treatment (20 and 50 mg) and placebo (*p < 0.05 and **p < 0.01).

The efficacy of the model in disrupting sleep is apparent when the whole night sleep variables from the baseline and placebo nights are compared. Under placebo, there was a significant increase in LPS (9.60 min; *p* = 0.006), SOL (5.18 min; *p* = 0.018), WASO (31.86 min, *p* = 0.002), and duration of wake (40.54 min; *p* = 0.001). There were also significant reductions in TST (47.64 min; *p* = 0.003), SE (9.92%; *p* = 0.003), REM duration (24.36 min; *p* = 0.004) and REM as a percentage of TST (3.91%; *p* = 0.025). These effects of the phase advance model were most pronounced in the first third of the night (see Table 1b). Spectral analysis of the EEG showed the effects of the model were most pronounced during the first third of the night where SWA was reduced and beta activity enhanced in NREM (Fig. 3). Computed over the entire night, there was no marked effect of the phase advance model on EEG power spectra.

### Effects of JNJ-48816274 on sleep initiation, continuity and duration

Both sleep initiation and continuity were significantly improved under JNJ-48816274 compared to placebo. LPS was significantly reduced (*p* < 0.0001) as were both SOL (*p* < 0.0001) and duration of wake computed over the sleep period (*p* < 0.01). There was, consequently, a significant increase in SE (*p* = 0.0215) for both 20 mg (p = 0.0148) and 50 mg (*p* = 0.0165) compared to placebo. There was a significant effect of treatment on whole night TST (*p* = 0.0215) which increased by 27.6 min with 20 mg (*p* = 0.0074) and 27.1 min with 50 mg (*p* = 0.0083) compared to placebo. Increased TST was also observed during the first third of the night under both 20 and 50 mg (*p* < 0.0001) compared to placebo. The number of night awakenings in the first third of the night was significantly reduced (*p* = 0.0122) under both dose levels compared to placebo.

### Effects of JNJ-48816274 on sleep structure and architecture

Across the 8-h sleep period, treatment affected REM sleep such that its duration (min) (*p* = 0.0213) and contribution to TST (REM%) (*p* < 0.05) were significantly increased and REM latency was reduced (*p* < 0.0001) for both doses compared to placebo. Specifically, when the data were analysed per thirds of the night, there was a significant increase in REM as a percentage of TST for the first (*p* < 0.01) and second (*p* < 0.05) third of the night under both 20 and 50 mg compared to placebo.

For NREM sleep, in the first third of the night, there was a significant increase in the duration of NREM (*p* = 0.0001) and specifically N2 (*p* = 0.0037) and N3 (*p* = 0.0004) under both treatments compared to placebo. By contrast, during the first third of the night with both doses there was a reduction in the duration of wake (*p* < 0.0001) and for N1 both the duration (*p* = 0.0109) and percentage of TST (*p* = 0.0002) reduced. In the second third of the night, for N3 both the duration (min) (*p* = 0.0243) and percentage of TST (*p* = 0.0103) were significantly reduced under both doses; N3 duration was significantly reduced in the final third of the night (*p* = 0.0456) for the 20 mg condition.

Quantitative analysis of the EEG signal for the whole night (Fig. 3) revealed there were no significant changes in NREM or REM for the individual 1 Hz or eight spectral bands under the two doses. However, during NREM in the first third of the night, there were significant increases in SWA and decreases in beta activity under treatment compared to placebo. During the second and final thirds of the night, there was a significant reduction in SWA under both doses compared to placebo.

### Subjective assessments of sleep

**Fig. 4:**
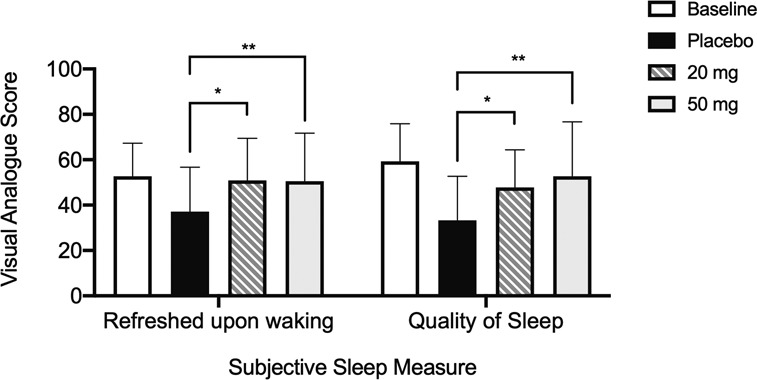
Subjective assessments of sleep measured by visual analogue scales in the Subjective Quality of Sleep Questionnaire (SQSQ) following the baseline and three treatment nights. White bars indicate baseline, black bars indicate placebo, hatched bars indicate 20 mg and grey bars indicate 50 mg. Significant differences between placebo and JNJ-48816274 treatment are indicated by *p < 0.05 and **p < 0.01.

Analysis of the SQSQ upon awakening (Fig. 4) revealed that the model induced a reduction in quality of sleep and feeling refreshed upon awakening. On treatment nights there was a significant effect on how refreshed participants felt upon awakening (*p* = 0.016) with greater refreshment after 20 (*p* < 0.05) and 50 mg (*p* < 0.01) compared to placebo. Self reported quality of sleep was significantly improved (*p* = 0.0005) with both 20 (*p* < 0.05) and 50 mg (*p* < 0.01) in comparison to placebo. The subjective assessment of time taken to fall asleep was reduced under 50 mg (*p* = 0.0206) and 20 mg (*p* = 0.07) compared to placebo (*p* < 0.01). There was a significant change in estimated total time spent asleep (*p* = 0.0221), which increased under both 20 and 50 mg (*p* < 0.05) compared to placebo.

The LSEQ revealed that ease of getting to sleep significantly improved (*p* = 0.0002) under both 20 (*p* = 0.0011) and 50 (*p* = 0.0001) mg compared to placebo. Participants found it significantly easier than usual to fall asleep (*p* < 0.0001) under 20 (*p* = 0.0002) and 50 (*p* < 0.0001) mg compared to placebo, and also fell asleep quicker than usual (*p* < 0.0001) with both 20 (*p* = 0.0003) and 50 (*p* < 0.0001) mg. With the LSEQ, quality of sleep was also significantly improved (*p* = 0.0002) with both doses (20 mg, *p* = 0.0051; 50 mg, *p* < 0.0001) with sleep being significantly more restful (*p* = 0.0001; 20 mg, *p* = 0.009; 50 mg, *p* < 0.0001) with fewer periods of wakefulness (*p* = 0.0039; 20 mg, *p* = 0.0097; 50 mg, *p* = 0.0017).

### Psychometric test battery: residual effects of treatment

Significant effects of treatment were observed only for subjective assessments (Supplementary Table 1). For the Bond & Lader questionnaire, compared to placebo, alertness was significantly greater under 20 (*p* < 0.01) and 50 mg (*p* = 0.012) at 8.75 h but not 10.25 h post dose, and contentedness was enhanced under both 20 and 50 mg at both 8.75 (*p* < 0.01, *p* < 0.05) and 10.25 h (*p* < 0.05) timepoints. The KSS assessment at the start of the test battery 8.75 h after dosing revealed that subjective sleepiness upon awakening was reduced under 20 (*p* < 0.01) and 50 (*p* < 0.01) mg compared to placebo.

### Adverse events

During the course of the trial, 28 treatment emergent adverse events (TEAEs) were reported for placebo (*n* = 11), 20 mg (*n* = 6) and 50 mg (*n* = 11) treatments. TEAEs of the nervous system included headache (*n* = 2 placebo, *n* = 1 for 50 mg) and somnolence immediately after dosing (*n* = 1 placebo, *n* = 3 for 50 mg), and under the psychiatric disorders classification, abnormal dreams were reported (*n* = 1 for 20 mg, *n* = 2 for 50 mg). Additional AEs reported included those related to the cannulation process (*n* = 2 placebo, *n* = 3 for 50 mg), musculoskeletal (*n* = 2 placebo, n = 1 for 50 mg), infection (*n* = 1 placebo, *n* = 1 for 20 mg), injury (*n* = 2 for 20 mg), respiratory (*n* = 1 placebo, *n* = 1 for 50 mg), gastrointestinal (*n* = 1 for 20 mg) and skin (*n* = 2 placebo). All TEAEs were considered mild except for a moderate infection in the placebo condition. All events of somnolence and abnormal dreams were considered “very likely” related to treatment.

## Discussion

Here we report on the impact of two doses of JNJ-48816274, a selective, high-affinity 2-SORA, on the timing, structure and quality of sleep initiated in the wake maintenance zone. Both 20 and 50 mg doses, in comparison to placebo, rapidly induced and maintained sleep, improved perceived sleep quality, and had minimal impact on “next day” waking performance. There was minimal difference between the doses on the variables measured.

The phase advance model successfully disrupted the duration, structure and initiation of sleep, as observed under placebo compared to baseline. These alterations of sleep, including an increased time awake, increased time to fall asleep, and reduction in TST and REM, were particularly apparent in the first third of the night, i.e., when sleep is scheduled during the wake maintenance zone. By comparison, JNJ-48816274 counteracted the disruptive effect of the model through improvements in the initiation and maintenance of sleep as indicated by a reduced LPS, SOL, and time awake, and increased SE. TST was significantly enhanced, in particular during the first third of the night where there was a ~50% increase. In the first third of the night, there was an increase in REM, N2 and N3 and SWA, with a corresponding reduction in wake, N1 and beta activity. This impact of JNJ-48816274 on sleep initiation, maintenance and duration in a phase advance model has also previously been observed for GABA-ergic hypnotics [19, 20] and exogenous melatonin (0.5–10 mg) (e.g. [35]).

The observed impact of JNJ-48816274 on sleep is consistent with previous observations with the 2-SORA seltorexant and DORAs. The DORA SB-649868, administered to healthy participants undergoing a traffic noise model of sleep disruption, increased TST and REM duration and decreased LPS, WASO and REM latency [36]. Similar effects on TST, LPS and REM have been observed with seltorexant in both pre-clinical models [15] and clinical populations [37, 38]. The current observed increase in sleep onset REM episodes was previously observed for DORAs [36], and may represent a general effect of orexin antagonism.

In the current study, quantitative analysis of the EEG signal for the whole night revealed no impact of either JNJ-48816274 dose on NREM or REM. These effects are similar to what was observed with DORAs (e.g. [36, 39]) but in contrast to the traditional GABA modulating sleep drugs (e.g. zopiclone, zolpidem, benzodiazepines) which have signature effects on the sleep EEG including a reduction in delta and theta activity, and enhanced sigma activity [40].

Overall, in line with previous work in ORAs (e.g. [37–39]), JNJ-48816274 was well tolerated with only 28 TEAEs (*n* = 11 placebo) reported throughout the trial. Only the events of somnolence immediately after study drug administration and abnormal dreams were considered to be very likely related to treatment and these were classified as mild. For safety reasons, only male participants were studied but we do not anticipate responses in females to be different.

The observed effects on sleep by JNJ-48816274 in a phase advance model likely arises from its ability to weaken the wake-promoting signal, which peaks in the early evening hours and coincides with the peak in the circadian rhythm of expected brain release concentration of orexin [41]. Populations that may benefit from hypnotics targeting the orexin wake-promoting system include patients with delayed sleep phase disorder (DSPD), a circadian rhythm sleep–wake disorder. DSPD patients have circadian clocks with later timing and thus may struggle with sleep initiation if they have a desired bedtime or attempt sleep at an earlier/advanced circadian phase, during their wake maintenance zone [42, 43]. In addition, patients with major depressive disorder exhibit elevated CSF orexin levels and reduced orexin level amplitude across the 24 h day compared to control participants [44] and as such, antagonising the orexin signal could be beneficial for those patients experiencing sleep disturbance. Night shift workers experiencing misalignment between their circadian system and the desired sleep/wake schedule with short duration, poor quality daytime sleep, may also benefit from orexin antagonists [45].

In summary, we have demonstrated that JNJ-48816274 is well tolerated and can effectively initiate and maintain sleep initiated in the wake maintenance zone with no residual effects upon awakening. Furthermore, we have shown that positive effects on sleep initiation and maintenance do not require antagonism of both orexin receptors; antagonism of the OX2R is sufficient.

## Funding and disclosure

Janssen Research and Development, a division of Janssen Pharmaceutical N.V., funded the research and provided the Investigational Medicinal Product. DJD served or serves as a paid consultant to and/or received research support from F. Hoffmann- La Roche Ltd, Pfizer Inc, Eli Lilly and Company, Novo Nordisk A/S, Ono Pharma UK Ltd, Janssen Research & Development LLC, Eli Lilly and Company, and GW Pharma, Phillips Lighting, H Lundbeck A/S, Merck Inc, Vanda Pharmaceuticals, Cephalon Inc, Servier, UCB, Procter & Gamble, Ferring Pharmaceuticals A/S. The other authors have nothing to disclose

## Supplementary information

Supplementary Table 1

CONSORT 2010 Flow Diagram
