# Supplementary material for: Effects of the selective orexin-2 receptor antagonist JNJ-48816274 on sleep initiated in the circadian wake maintenance zone: a randomised trial
Source: Neuropsychopharmacology. 2021 Oct 9;47(3):719–27. doi: 10.1038/s41386-021-01175-3 (PMC8782905; doi:10.1038/s41386-021-01175-3)
Supplement: Supplementary file 1 — Supplementary Table 1 [file 41386_2021_1175_MOESM1_ESM.pdf]

Supplementary Table 1

Psychometric Variable		Estimated difference vs Placebo (p-value)					
	Variable	20 mg			50 mg		
		Difference	95% Confidence Intervals	Contrast p value	Difference	95% Confidence Intervals	Contrast p value
Karolinska Sleepiness Scale	Scale at 8.75 h post-dose	-1.00	(-1.71,-0.29)	0.006	-1.17	(-1.88,-0.46)	0.001
Bond & Lader VAS	Derived Factor - Alert - 8.75 h post dose	-45.35	(-78.37, -12.32)	0.008	-42.6	(-75.62, -9.57)	0.012
	Derived Factor - Contented - 8.75 h post dose	-22.13	(-37.66, -6.60)	0.006	-17.38	(-32.92, -1.85)	0.029
	Derived Factor - Calm - 8.75 h post dose	-2.11	(-11.20, 6.99)	0.64	-5.67	(-14.76, 3.43)	0.22
Body Sway	A95% - Eyes Open	-0.084	(-0.215 , 0.047)	0.2009	-0.019	(-0.153 , 0.114)	0.7696
	Length of COP - Eyes Open	-1.141	(-2.393 , 0.112)	0.0727	-1.188	(-2.440 , 0.064)	0.0621
	A95% - Eyes Closed	-0.336	(-0.809 , 0.136)	0.1566	-0.155	(-0.629 , 0.319)	0.51
	Length of COP - Eyes Closed	-0.597	(-2.215 , 1.021)	0.4562	-1.53	(-3.158 , 0.097)	0.0644
Simple Reaction Time	Total Reaction Time (msec)	-14.78	(-39.947 , 10.386)	0.2404	-13.749	(-38.916 , 11.417)	0.2741
Choice Reaction Time	Total Reaction Time (msec)	-12.087	(-41.858 , 17.684)	0.4144	-4.661	(-34.432 , 25.110)	0.7519
Verbal N-back 1	% correct responses	0.617	(-0.737 , 1.972)	0.3602	-0.309	(-1.663 , 1.046)	0.6457
	Mean response time for correct responses	-16.213	(-56.949 , 24.524)	0.4235	-29.771	(-70.508 , 10.966)	0.1464
Verbal N-back 2	% correct responses	1.235	(-0.807 , 3.276)	0.2271	0.772	(-1.270 , 2.814)	0.4471
	Mean response time for correct responses	1.453	(-57.760 , 60.666)	0.9604	2.759	(-56.455 , 61.972)	0.925
Verbal N-back 3	% correct responses	-0.232	(-3.153 , 2.689)	0.8727	1.003	(-1.918 , 3.924)	0.4893
	Mean response time for correct responses	-12.482	(-65.332 , 40.368)	0.6337	-27.096	(-79.946 , 25.754)	0.3042

Supplementary Table 1. Psychometric variables (mean + 95% confidence intervals) and differences between JNJ-48816274 treatment (20 and 50 mg) and placebo.
