# Supplementary material for: Effects of the selective orexin-2 receptor antagonist JNJ-48816274 on sleep initiated in the circadian wake maintenance zone: a randomised trial
Source: Neuropsychopharmacology. 2021 Oct 9;47(3):719–27. doi: 10.1038/s41386-021-01175-3 (PMC8782905; doi:10.1038/s41386-021-01175-3)
Supplement: Supplementary file 2 — CONSORT 2010 Flow Diagram [file 41386_2021_1175_MOESM2_ESM.doc]

**
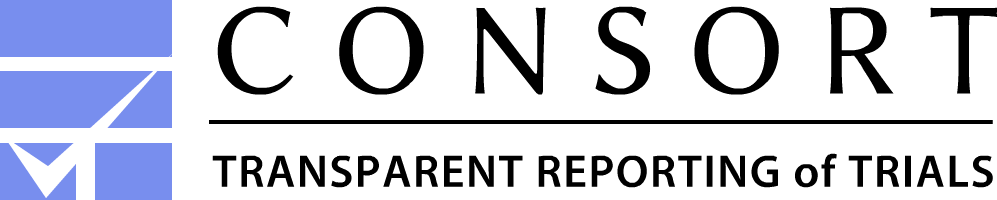
**

**CONSORT 2010 Flow Diagram**

**Allocation**

**Analysis**

**Follow-Up**

**Enrollment**

Assessed for eligibility (n = 125) )

Excluded (n = 107)

♦  Not meeting inclusion criteria (n = 99)

♦  Declined to participate (n = 8)

Lost to follow-up (n = 0)

Discontinued intervention (n = 0)

Allocated to intervention (n = 18)

♦ Received allocated intervention (n = 18)

♦ Did not receive allocated intervention (n = 0)

Analysed (n = 18)
♦ Excluded from analysis (n = 0)

Randomized (n = 18)
